# Transcription-based comparison of *Aggregatibacter actinomycetemcomitans* or *Porphyromonas gingivalis*-induced experimental periodontitis

**DOI:** 10.1128/spectrum.01678-25

**Published:** 2026-01-08

**Authors:** Emiliano Vicencio, Mauro Cortez, Luis González-Osuna, Samanta Melgar-Rodríguez, Carolina Rojas, Vanessa Campos-Bijit, Adolfo Rojas, Ignacio Pezoa-Soto, Vinicius Maracaja-Coutinho, Alberto J. M. Martin, Juan Pablo Cardenas, Rolando Vernal, Cristian Cortez

**Affiliations:** 1 Escuela de Tecnología Médica, Facultad de Ciencias, Pontificia Universidad Católica de Valparaíso 28047, Valparaíso, Chile; 2 Laboratorio de Biología Periodontal, Facultad de Odontología, Universidad de Chile 14655, Santiago, Chile; 3 Laboratory of Nanobiomaterials, Research Institute of Dental Sciences, Faculty of Dentistry, Universidad de Chile 14655, Santiago, Chile; 4 Unidad de Genómica Avanzada - UGA, Advanced Center for Chronic Diseases - ACCDiS, Facultad de Ciencias Químicas y Farmacéuticas, Universidad de Chile 14655, Santiago, Chile; 5 Centro de Biología Celular y Biomedicina (CEBICEM), Facultad de Medicina y Ciencia, Universidad San Sebastián 28088, Santiago, Chile; 6 Laboratorio de Redes Biológicas, Fundación Ciencia & Vida, Santiago, Chile; 7 Laboratório de Medicina e Saúde Pública de Precisão - MESP2, Instituto Gonçalo Moniz, Fundação Oswaldo Cruz, Fiocruz 14655, Salvador, Brazil; 8 Escuela de Ingeniería, Facultad de Ingeniería, Universidad San Sebastián 28088, Santiago, Chile; 9 Centro de Genómica y Bioinformática, Facultad de Ciencias, Ingeniería y Tecnología, Universidad Mayor 28084, Santiago, Chile; 10 Escuela de Biotecnología, Facultad de Ciencias, Ingeniería y Tecnología, Universidad Mayor 28084, Santiago, Chile; 11 Departamento de Odontología Conservadora, Facultad de Odontología, Universidad de Chile 14655, Santiago, Chile; 12 Escuela de Tecnología Médica, Facultad de Ciencias, Universidad San Sebastián 28088, Santiago, Chile; University of Dundee, Dundee, United Kingdom

**Keywords:** experimental periodontitis, dysbiotic biofilm, bulk RNA-seq, transcriptome, gene regulatory network, master regulator transcription factors

## Abstract

Periodontitis is a chronic inflammatory disease caused by a dysregulated immune response against the subgingival dysbiotic biofilm. Among the Gram-negative bacteria detected in the infected periodontium, *Aggregatibacter actinomycetemcomitans* (*Aa*) and *Porphyromonas gingivalis* (*Pg*) are strongly associated with the most destructive forms of periodontitis. Given their phylogenetic divergence and distinct virulence potential, we performed a comparative transcriptomic analysis in a murine experimental periodontitis model induced by separate direct inoculations of *Aa* and *Pg*. After confirming periodontal destruction, a massive RNA sequencing (RNA-seq) was conducted on the palatal mucosa, which lines and forms part of the tissues affected by periodontitis. Our analysis identified 91 differentially expressed genes (DEGs) in response to *Aa* and 119 DEGs in response to *Pg*, with only 22 shared DEGs, 12 of which were associated with the humoral immune response. Comparative analysis revealed eight distinct co-expression modules, each exhibiting differential gene representation and expression patterns. Notably, Module 4, linked to the immune response, displayed a similar expression profile for both bacteria. Additionally, we constructed gene regulatory networks (GRNs) from transcriptomic data and identified a subnetwork comprising 8 clusters, 54 nodes, 17 transcription factors, and 53 regulatory interactions. Finally, the transcription factors *FOS*, *JUN*, *RELA*, *TP53*, *EGR1*, and *NFκB1* were identified as master regulators (MR-TFs) of inoculation-induced murine periodontitis and were conserved in human regulatory networks.

**IMPORTANCE:**

Periodontitis is the most common osteolytic disease in humans, significantly affecting oral health and worsening various systemic inflammatory conditions. Specific bacteria, such as *A. actinomycetemcomitans* and *P. gingivalis*, are frequently found in severe cases. This highlights the need to implement advanced methodologies to understand their underlying pathogenic mechanisms. We used massive RNA-seq to analyze, for the first time, the complete palatal mucosa of animals affected by, or not affected by, experimental periodontitis induced by the most virulent serotypes of both bacterial species. Our findings reveal that these bacteria explore distinct molecular pathways to induce disease. Despite their phylogenetic differences and distinctive virulence factors, *A. actinomycetemcomitans* and *P. gingivalis* activate common transcriptional regulators that promote periodontitis progression, suggesting conserved molecular mechanisms underlying periodontal destruction. These results provide valuable information for developing therapeutic strategies to modulate these regulatory nodes and improve treatment outcomes in periodontitis and other related inflammatory conditions.

## INTRODUCTION

Periodontitis, the most prevalent osteolytic pathology in humans, is a chronic, non-communicable inflammatory disease that progressively destroys the periodontium (1). Its clinical manifestations include gingival inflammation, clinical attachment loss, alveolar bone resorption, periodontal pocket formation, and ultimately, tooth loss (2, 3). Periodontitis is closely linked to and can influence the course of other systemic inflammatory conditions, including rheumatoid arthritis, osteoporosis, type II diabetes mellitus, Alzheimer’s disease, cardiovascular disorders, and adverse pregnancy outcomes (4). The primary etiological factor in periodontitis is the persistent challenge caused by a dysbiotic subgingival biofilm, which triggers a deregulated host immune response that drives the disease phenotype (5).

Certain bacterial species with pathogenic potential have been associated with the onset, progression, and severity of periodontitis. While these bacteria can directly damage periodontal tissues, the primary driver of irreversible destruction is the host immune response elicited against them (6). Among the most strongly implicated periodontopathogens are *Porphyromonas gingivalis* (*Pg*), an anaerobic, asaccharolytic Gram-negative rod, and *Aggregatibacter actinomycetemcomitans* (*Aa*), a capnophilic, facultative anaerobic Gram-negative rod (5, 7, 8). Their association with destructive periodontitis is supported by their virulence properties, their recurrent detection in diseased periodontium, and their infrequent detection in periodontally healthy tissues (9, 10). Based on capsular antigen heterogeneity, *Pg* is classified into six serotypes (K1–K6), which can be divided into invasive (encapsulated) and non-invasive (non-encapsulated) strains, with serotype K1 being the most virulent (11). In turn, *Aa* is classified into seven serotypes (a–g) based on its lipopolysaccharide (LPS) composition, with serotype b being most commonly associated with severe periodontitis (12, 13).

Despite their shared association with periodontal destruction, *Pg* and *Aa* are phylogenetically unrelated (14, 15) and exhibit distinct virulence mechanisms that enable them to colonize the gingival sulcus, infiltrate the junctional epithelium, invade and spread through connective tissues, modulate host immune responses, and induce pathological alveolar bone loss, the hallmark of periodontitis (6, 16–18). While some molecular mechanisms underlying their pathogenicity have been characterized, a comprehensive comparative analysis of the specific genes, signaling pathways, and host gene regulatory networks (GRNs) involved in their pathogenic strategies remains incomplete. This knowledge gap limits the development of targeted therapeutic approaches to mitigate disease progression.

Given that the palatal mucosa is an essential component of the protective periodontium, responsible for safeguarding the alveolar bone and periodontal ligament against microbial aggression, our previous studies (19) demonstrated that transcriptomic analysis (Bulk RNA-seq) of the entire palatal mucosa allows for a comprehensive characterization of the molecular changes associated with periodontal inflammation in a murine model of ligature-induced periodontitis. In that work, we identified 408 differentially expressed genes (DEGs) primarily associated with the host immune response, as well as 26 master regulatory transcription factors (MR-TFs) involved in periodontitis pathogenesis. However, a key limitation of the ligature model is that *Pg* and *Aa* are not native constituents of the murine oral microbiota and therefore are not implicated in the development of murine periodontitis (20, 21). Consequently, although the ligature model effectively mimics the inflammatory damage observed in human periodontitis, it does not fully capture the variations in the immune response elicited by these specific pathogens. To address this limitation, we developed an experimental periodontitis model in which the most virulent serotypes of *Pg* and *Aa* were directly inoculated into murine periodontal tissues.

Given their distinct phylogenetic backgrounds, we hypothesized that *Pg* and *Aa* would induce periodontitis through distinct transcriptional pathways and regulatory mechanisms. Here, we conducted a comparative transcriptomic analysis of periodontal lesions in mice using high-throughput RNA-seq. This approach enabled the identification of gene expression patterns, signaling pathways, and transcriptional regulators that govern the pathogenic strategies of these bacteria.

## RESULTS

### Periodontitis induction and assessment of alveolar bone loss

**Fig 1:**
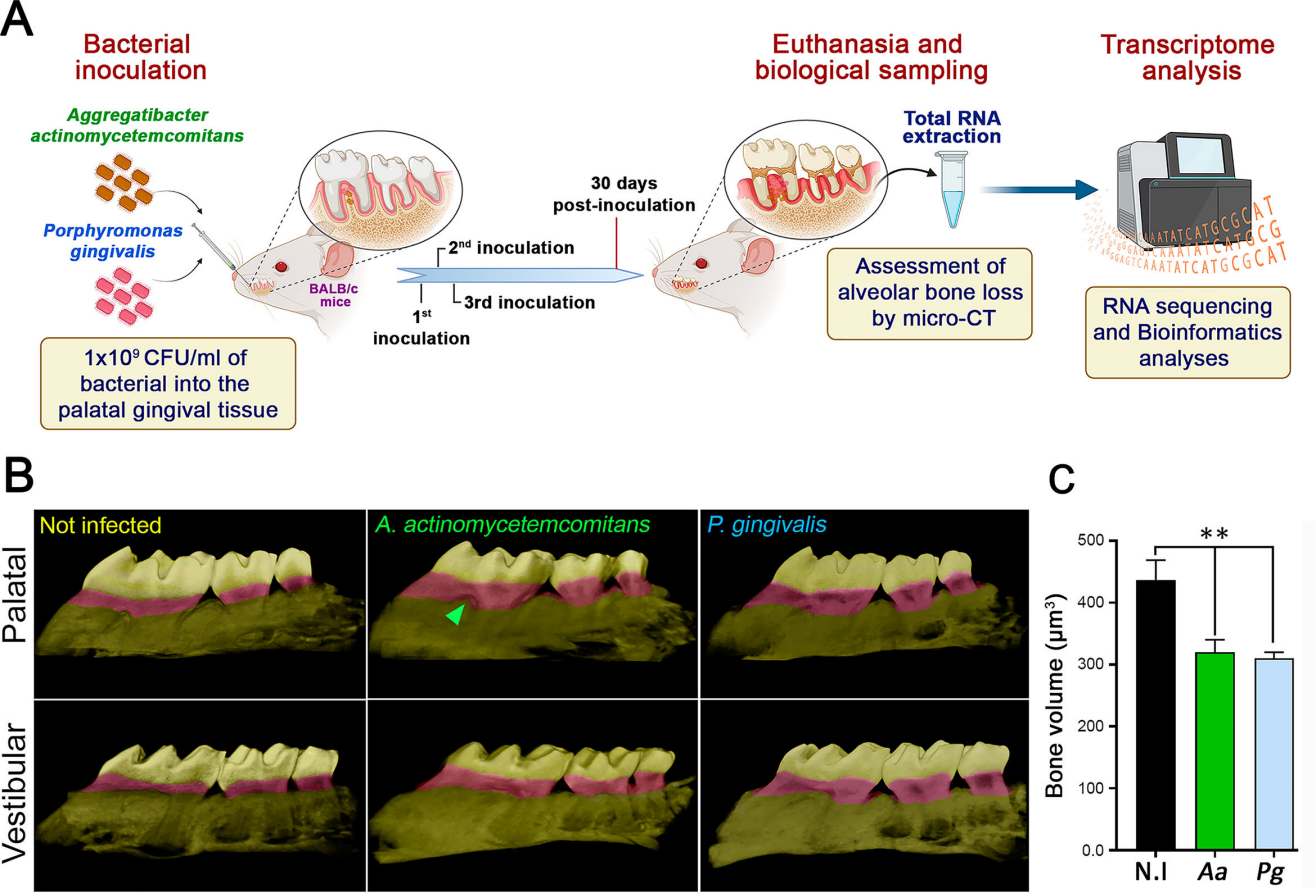
Experimental periodontitis and alveolar bone resorption assessment. (**A**) Schematic representation of bacteria inoculation-induced periodontitis. After 30 days post-induction, the periodontal tissues were extracted for analysis in this study. (**B**) Representative 3D micro-CT images of the maxillae from mice with periodontitis caused by bacterial inoculation and healthy control mice that were not infected. The ROI described in Materials and Methods, in which bone loss analyses were performed, is highlighted in purple. The green arrowhead indicates a palatine-furcation lesion in the first molar. (**C**) Alveolar bone volume quantified by micro-CT in maxilla specimens. The data are displayed as mean ± SD for bone-volume area (*n* = 5); **P < *0.01*.

We used a murine model of experimental periodontitis to identify genes and signaling pathways activated by *Aa* and *Pg* to drive the disease. This model is based on the direct inoculation of these bacteria into periodontal tissues, as illustrated in Fig. 1A and detailed in Materials and Methods. Since alveolar bone loss is a hallmark of periodontitis, we used micro-computed tomography (micro-CT) to analyze the maxillary molars of infected and uninfected mice. The bone volume measurements (μm^3^) in infected mice after 3D reconstruction (Fig. 1B) showed a significantly (*P* ˂ 0.01) higher alveolar bone loss compared to the uninfected control mice (Fig. 1C). Periodontitis mice exhibited bone loss 30 days post-inoculation, specifically between the cement-enamel junction and the alveolar bone crest, as well as between the mesial surface of the first molar and the distal surface of the third molar (highlighted in purple in Fig. 1B). These results indicate that experimental periodontitis was successfully induced.

### Global expression patterns and DGEs analysis in experimental periodontitis induced by the inoculation of *A. actinomycetemcomitans* or *P. gingivalis*

**Fig 2:**
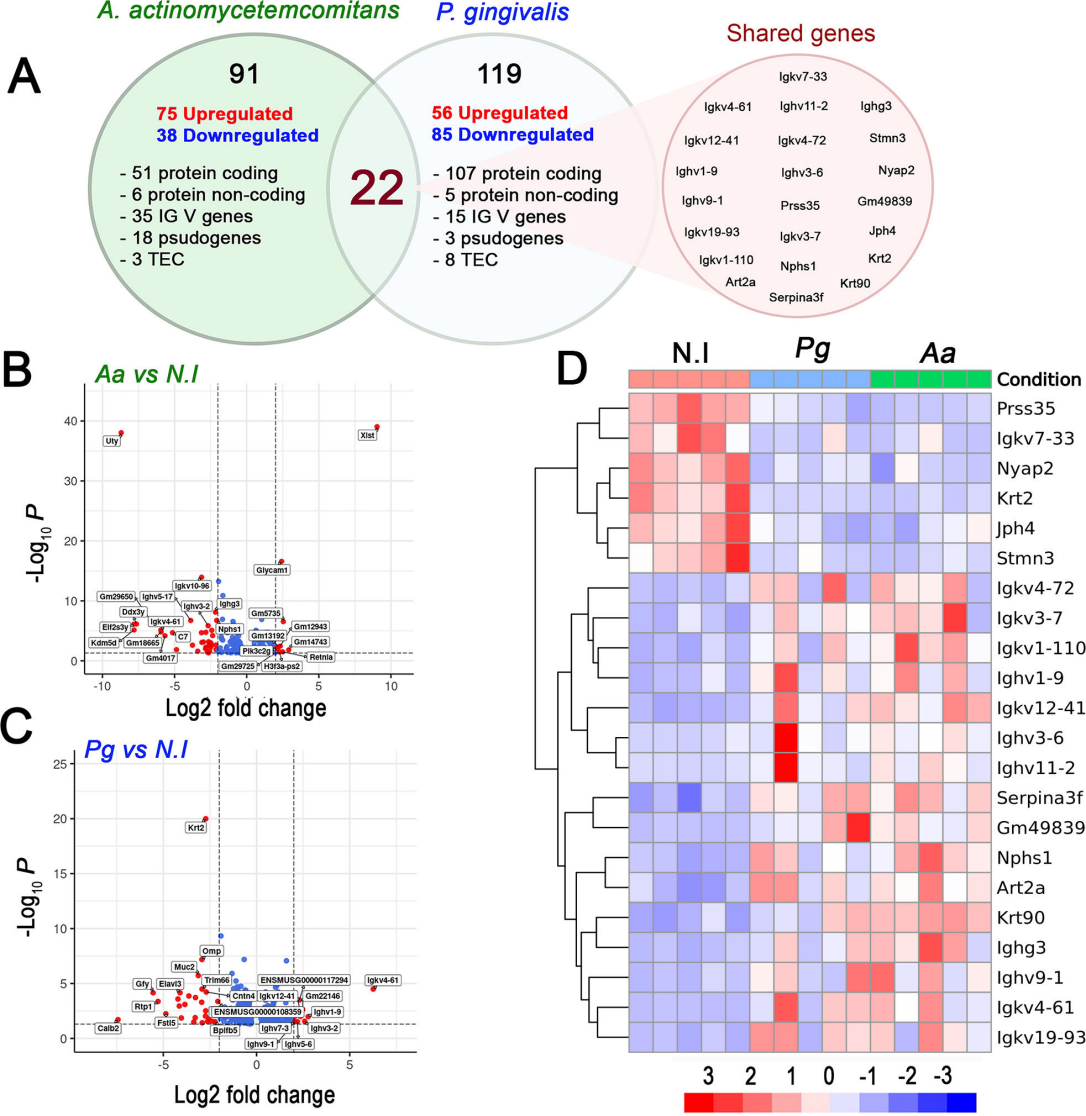
Transcriptomic analysis of mice with experimental periodontitis reveals mRNA expression changes induced by different bacteria. (**A**) Venn diagram showing the different types of differentially expressed (DE) genes in the palatal mucosa. The red circle indicates the 22 DE genes shared by both pathogens. (**B and C**) Volcano plots based on FPKM values show DE genes in the palatal mucosa of infected mice compared to uninfected mice. The plots are statistically significant (*P* < 0.05, Student’s *t*-test). (**D**) Heatmap from RNA-seq analysis showing the 22 differentially expressed coding genes in the palatal mucosa, shared by periodontitis models induced by *Aa* and *Pg*. An apparent clustering by condition is observed, highlighting the separation between the two infected groups and the uninfected control, and demonstrating marked transcriptomic differences associated with infection (*P* < 0.01, Student’s *t*-test).

**Fig 3:**
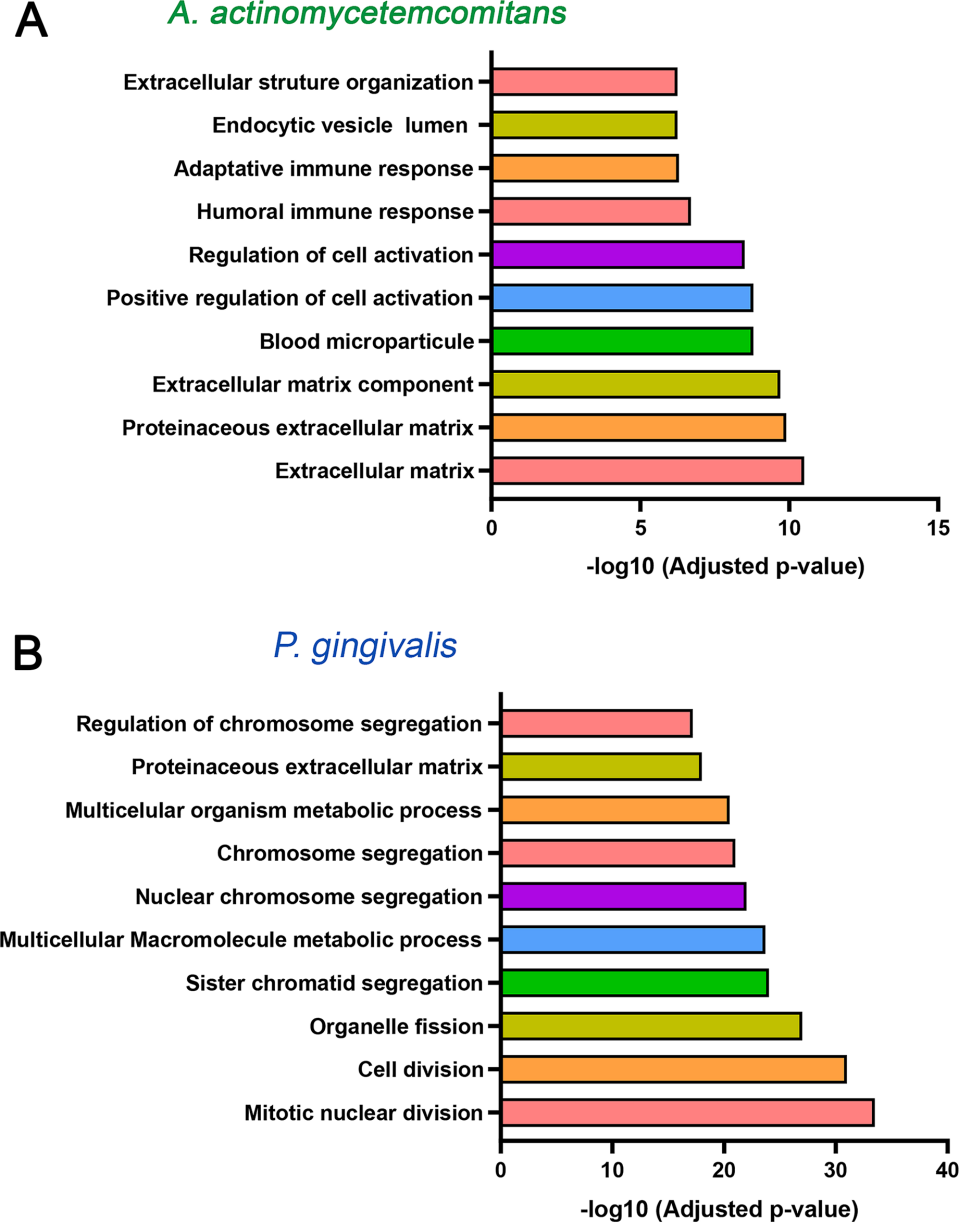
Transcriptomic analysis of mice with experimental periodontitis revealed distinct signaling pathways depending on the inoculated pathogen. Top 10 significantly enriched pathways of DE genes (*P* < 0.05, Student’s *t*-test) from the Elsevier pathway collection database. Pathways triggered by *Aa* (**A**) or *Pg* (**B**).

Subsequently, we performed RNA-Seq to examine transcriptional changes in the periodontal tissues of uninfected and periodontitis-affected mice. To achieve our goal, we extracted complete samples of oral palatal mucosa from all three experimental groups. This tissue was selected because the palatal mucosa is a specialized epithelial lining that covers the hard palate and part of the soft palate. It features a firm, keratinized surface that acts as an important mechanical and biological barrier against periodontal pathogens. The tissue architecture comprises various cell types that play significant roles in the periodontal response, including keratinocytes, melanocytes, Langerhans cells, fibroblasts, innate and adaptive immune cells, and bone cells, such as osteoblasts and osteoclasts. The integrity, inflammatory response, and defensive capacity of this mucosa are essential for managing periodontal damage. Therefore, analyzing this tissue provides a biologically relevant model for studying the cellular, immunological, and structural responses and mechanisms involved in the pathogenesis of periodontitis (22). Global transcriptional changes revealed 91 DGEs responding to *Aa* inoculation and 119 DGEs following *Pg* inoculation. For this analysis, only those genes that met a significance level of p.adj < 0.05 and a fold change > 1.0 in periodontitis mice compared to healthy mice were considered differentially expressed (Fig. 2A). According to these criteria, 75 upregulated and 38 downregulated genes were identified in response to *Aa* inoculation. In contrast, *Pg* inoculation resulted in 56 upregulated and 85 downregulated genes (Fig. 2A–C). Additionally, when comparing the experimental periodontitis group with healthy controls, the Venn diagram evidenced 22 shared DGEs. These genes are associated with the indirect regulation of T-cell apoptosis (*Art2a*); inflammation and tissue remodeling (*Prss35* and S*erpina3f*); cytoskeletal structure and function (*Nphs1*, *Stmn3*, *Krt2*, and *Krt90*); and neuronal morphogenesis and plasticity (*Prss35*, *Nyap2*, and *Jph4*). The most represented function, with 12 genes involved, was the humoral immune response through the DGEs encoding immunoglobulin heavy chains (*Igkv*, *Ighv*, and *Ighg*) (Red circle in Fig. 2A). Biological variability between samples was analyzed using heatmaps, which allowed the identification of hierarchical clusters and the expression levels of protein-coding DGEs in the different experimental groups (Fig. 2D). This approach facilitated clear differentiation of gene expression patterns between healthy mice and those with *Aa*- or *Pg*-induced periodontitis, specifically among the 22 shared genes, highlighted in red in the Venn diagram shown in Fig. 2A. Notably, these 22 DGEs shared by the *Aa*- and *Pg*-induced inoculation models showed concordant expression patterns across the two conditions. In particular, the genes *Prss35*, *IgKv7-33*, *Nyap2*, *Krt2*, *Jph4*, and *Stmn3* were consistently downregulated in both infection models compared with the uninfected control group, as shown in the upper left quadrant of Fig. 2D. In contrast, the genes *IgKv4-72*, *IgKv-7*, *IgKv1-110*, *Ighv1-9*, *IgKv12-41*, *Ighv3-6*, *Ighv11-2*, *Serpina3f*, *Gm49839*, *Nphs1*, *Art2a*, *Krt90*, *Ighg3*, *Ighv9-1*, *IgKv4-61*, and *IgKv19-93* were upregulated in both inoculation conditions compared to uninfected controls, as evidenced in the lower left quadrant of Fig. 2D. To further explore the functional pathways underlying the differential expression of these transcripts, we performed a Gene Ontology (GO) term enrichment analysis. This analysis highlighted an overrepresentation of functional terms related to extracellular matrix remodeling and function, immune response, and cellular activation in *Aa*-induced periodontitis (Fig. 3A). In contrast, *Pg*-induced periodontitis primarily activated signaling pathways related to cell division, nuclear mitotic events, and macromolecular metabolic processes. Additionally, although to a lesser extent, pathways associated with extracellular matrix protein synthesis were observed (Fig. 3B). Altogether, these results highlight distinct, yet consistent molecular responses triggered by *Aa* and *Pg*, indicating that each pathogen activates specific mechanisms of disease. Furthermore, the observed convergence among subsets of genes commonly regulated suggests that, despite their different virulence strategies, both bacteria engage core pathways central to the host’s inflammatory and immune responses.

### Gene co-expression networks during microbial inoculation-induced periodontitis

**Fig 4:**
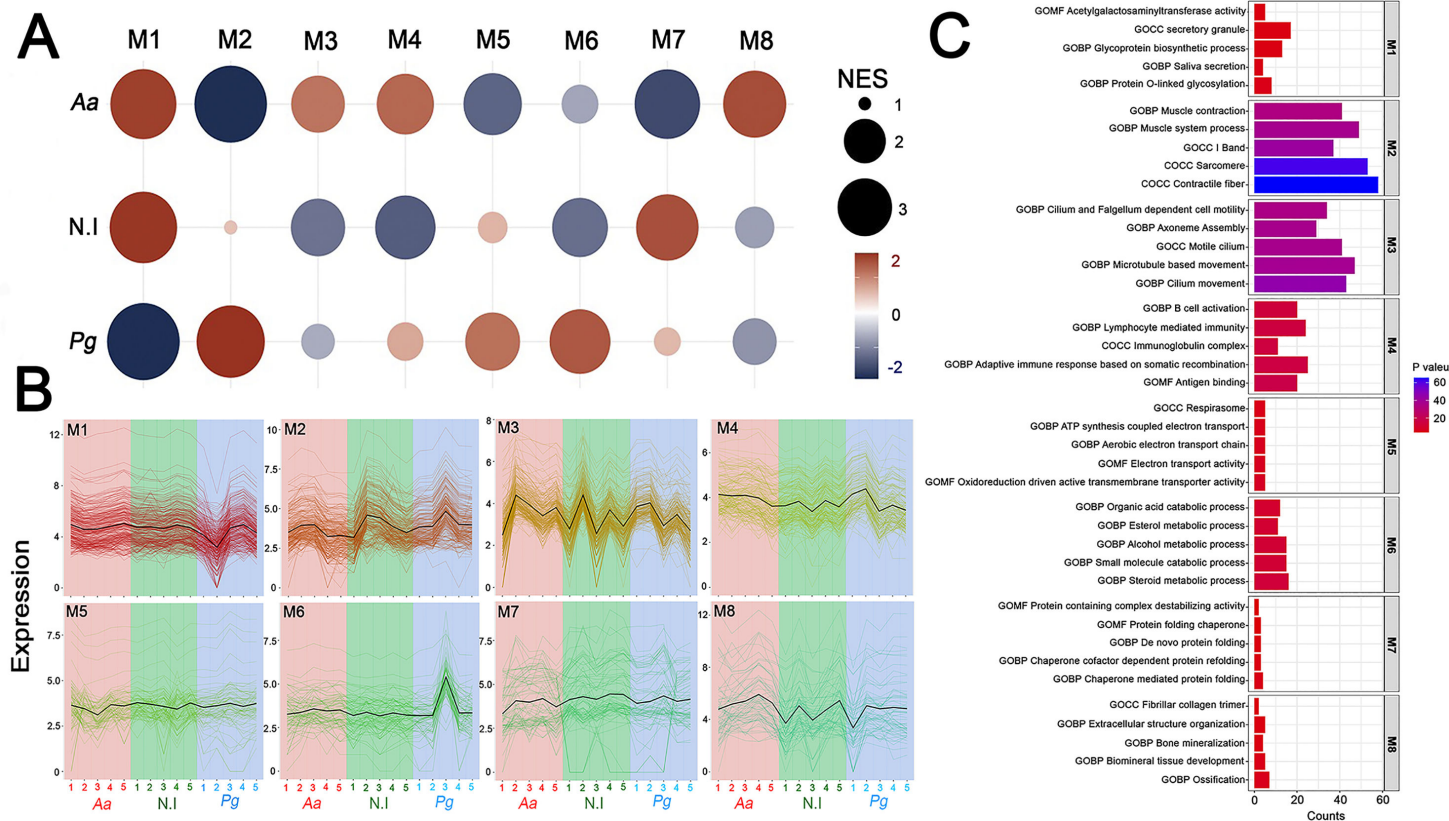
Co-expression module analysis during experimental periodontitis. (**A**) Gene set enrichment analyses that display the module activity. The size and color of the circle represent the normalized enrichment score (NES). From top to bottom, the figure shows the activities of Aa (the uninfected control) and Pg. (**B**) Profile plots of the eight modules. The expression levels of individual genes from each module are shown as colored lines. The black line represents the mean expression of all genes inside the module. The x-axis displays samples, colored by class: *Aa* in red, uninfected in green, and *Pg* in light blue. (**C**) Enriched horizontal bars representing each module’s top five biological processes, identified through gene set enrichment analysis (GSEA) using MSigDB (29) in CEMiTool.

**Fig 5:**
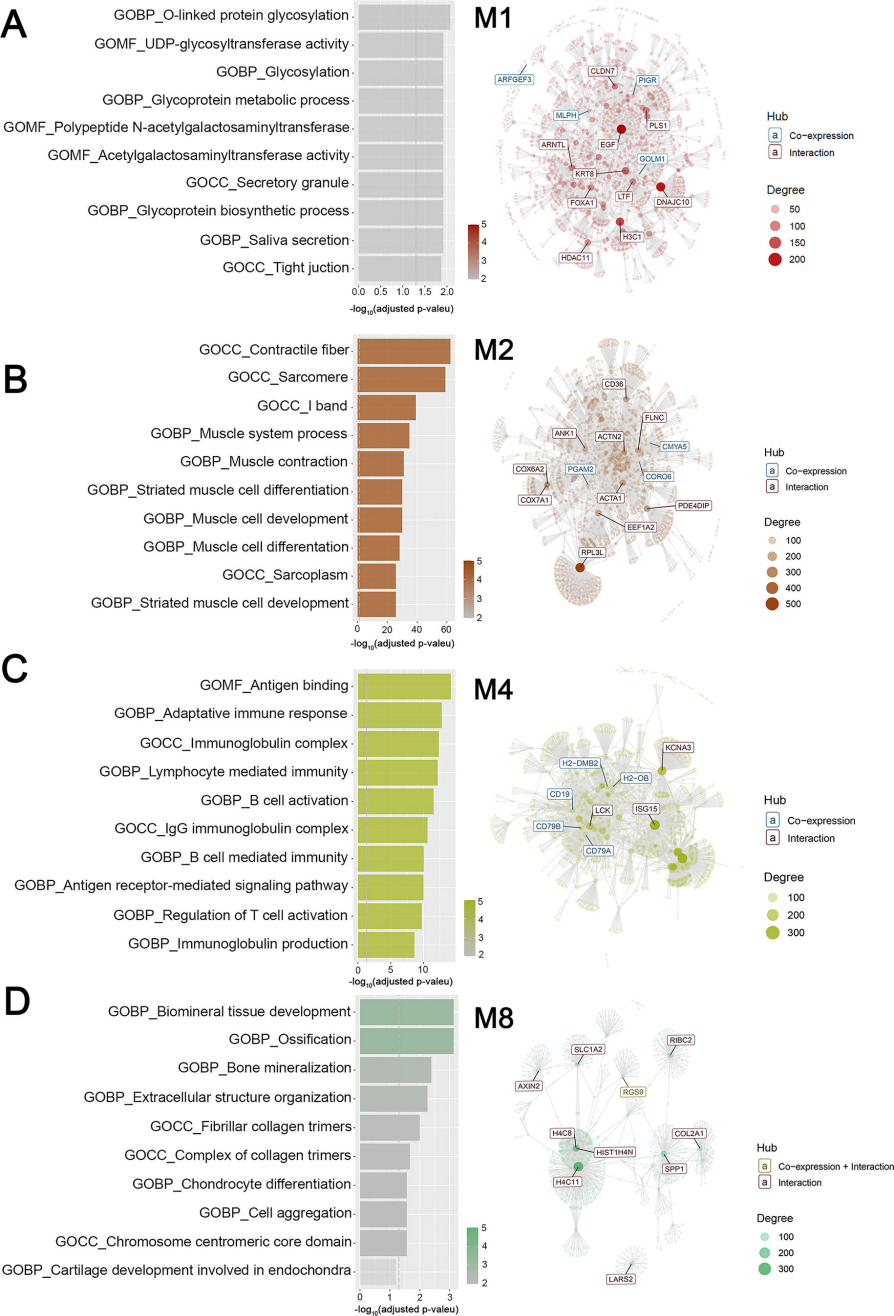
Signaling pathways and gene networks of the most interconnected co-expression modules. The bar graphs on the left display the top 10 functional terms with the highest overrepresentation. On the right, interaction plots highlight the core genes within each co-expression module. Overrepresentation analysis (−log10 adjusted *P*-value) was performed using gene set enrichment analysis from Msigdb (29) for modules M1 (**A**), M2 (**B**), M4 (**C**), and M8 (**D**) via the CEMiTool and String databases. The x-axis shows a ranking of pathways by significance. The vertical dashed gray line marks the threshold for an adjusted *P*-value of ≤0.05 using Student’s *t*-test. The interaction plots highlight the hub genes in modules M1, M2, M4, and M8, with node size reflecting their connectivity degree.

Modular gene co-expression networks provide a systems-level framework to understand the functional organization of the transcriptome. A gene co-expression network is defined as a set of genes connected based on the degree to which their expression levels are correlated across samples. Thus, two genes are considered “co-expressed” when their expression profiles vary similarly. This correlation structure enables the grouping of genes into highly interconnected modules that often correspond to shared biological processes, coordinated transcriptional regulation, or participation in common molecular pathways (23). Using this approach, we functionally classified genes in our data set and identified those with potential regulatory roles within each module (19, 24–26), which is crucial for understanding gene interactions and regulatory mechanisms in the pathology under investigation. For this purpose, we employed CEMiTool (27, 28), using as input a matrix of normalized gene expression counts that integrated the three experimental conditions: uninfected mice, *Aa*-infected mice, and *Pg*-infected mice. This data structure enabled the software to infer gene modules from joint variation in expression profiles, enabling direct comparison between the baseline transcriptome and the transcriptional changes induced by infection. Comparative evaluation of the three conditions revealed gene clustering into eight co-expression modules (M1–M8), which differed in both representation and expression levels (Fig. 4A). This can be observed through the varying sizes and colors of the circles (modules) in Fig. 4A and the heterogeneous dynamics of individual gene expression profiles across samples from the different experimental groups in Fig. 4B. The M1, M2, M3, and M4 modules were the most enriched, with 270, 236, 226, and 150 genes, respectively (Fig. 4A). The eight identified modules showed functional enrichment associated with diverse biological processes involved in glycoproteins biosynthesis-related structural and functional processes (M1); striated muscular system’s molecular structures and functions (M2); cytoskeleton’s role in cilia and flagella composition, assembly, and functionality in eukaryotic cell motility (M3); function and differentiation of lymphocytes during the adaptive immune response (M4); redox processes and mitochondrial energy production (M5); lipid and steroid enzymatic regulation and small molecule metabolism (M6); dynamic protein assembly processes, mitotic spindle regulation, and epidermal differentiation (M7); and bone metabolism, biomineralization, and skeletal tissue organization (M8) (Fig. 4C and 5 and Fig. S1). Among the modules analyzed, M4, associated with the humoral immune response, was the only one to exhibit a pattern of upregulation similar to that induced by *Aa* and *Pg* (red circles in Fig. 4A). In contrast, the remaining seven modules exhibited opposite co-expression patterns depending on the inoculating pathogen. For example, modules M1, M3, and M8 were upregulated in response to *Aa*, while M2, M5, M6, and M7 were downregulated (blue circles in Fig. 4A). Conversely, *Pg* displayed antagonistic behavior for these same modules. To ensure a comprehensive understanding of the gene co-expression patterns induced by *Aa* and *Pg*, we conducted an integrative analysis using CEMITool, which combined the normalized gene expression matrix (the same one shown in Fig. 4, including the three experimental conditions) with curated protein-protein interaction data to calculate gene correlations and construct a weighted co-expression network (network in Fig. 5 and Fig. S1). Epidermal Growth Factor (*EGF*), DnaJ Heat Shock Protein Family (*Hsp40*) Member C10 (*DNAJC10*), H3 Clustered Histone 1 (*H3C1*), Keratin 8 (*KRT8*), and Lactotransferrin (*LTF*) were identified as hub genes, that is, nodes with the highest number of connections in the M1 network (network in Fig. 5A). Ribosomal Protein L3 like (*RPL3L*), Eukaryotic Translation Elongation Factor 1 Alpha 2 (*EEF1A2*), Actin Alpha 1 and 2 (*ACTA1, ACTN2*), Skeletal Muscle (*ACTA1*), Filamin C (*FLNC*), Ankyrin 1 (*ANK1*), and Phosphodiesterase 4D Interacting Protein (*PDE4DIP*) were identified in the M2 network (Fig. 5B). According to its functional representation, the hub genes found in the M4 module were Cluster of Differentiation 19 molecule (*CD19*), Cluster of Differentiation 79a and 79b (*CD79a* and *CD79b*), LCK proto-oncogene, Src family tyrosine kinase (*LCK*), Potassium Voltage-Gated Channel Subfamily A Member 3 (*KCNA3*), and ISG15 ubiquitin-like modifier (*ISG15*). These genes are essential in innate and adaptive immune responses (network in Fig. 5C). At the same time, H4 Clustered Histone 11 (*H4C11*), H4 Clustered Histone 8 (*H4C8*), Secreted Phosphoprotein 1 (*SPP1*), Collagen Type II Alpha 1 Chain (*CL2A1*), Axin 2 (*AXIN2*), and RIB43A-like with coiled-coils protein 2 (*RIBC2*) were the most connected in M8 (network in Fig. 5D). The combined findings from Fig. 4 and 5 and Fig. S1 support the hypothesis that *Aa* and *Pg* could utilize distinct molecular pathways to reach similar pathological outcomes.

### Context-specific GRNs on inoculation-induced experimental periodontitis

**Fig 6:**
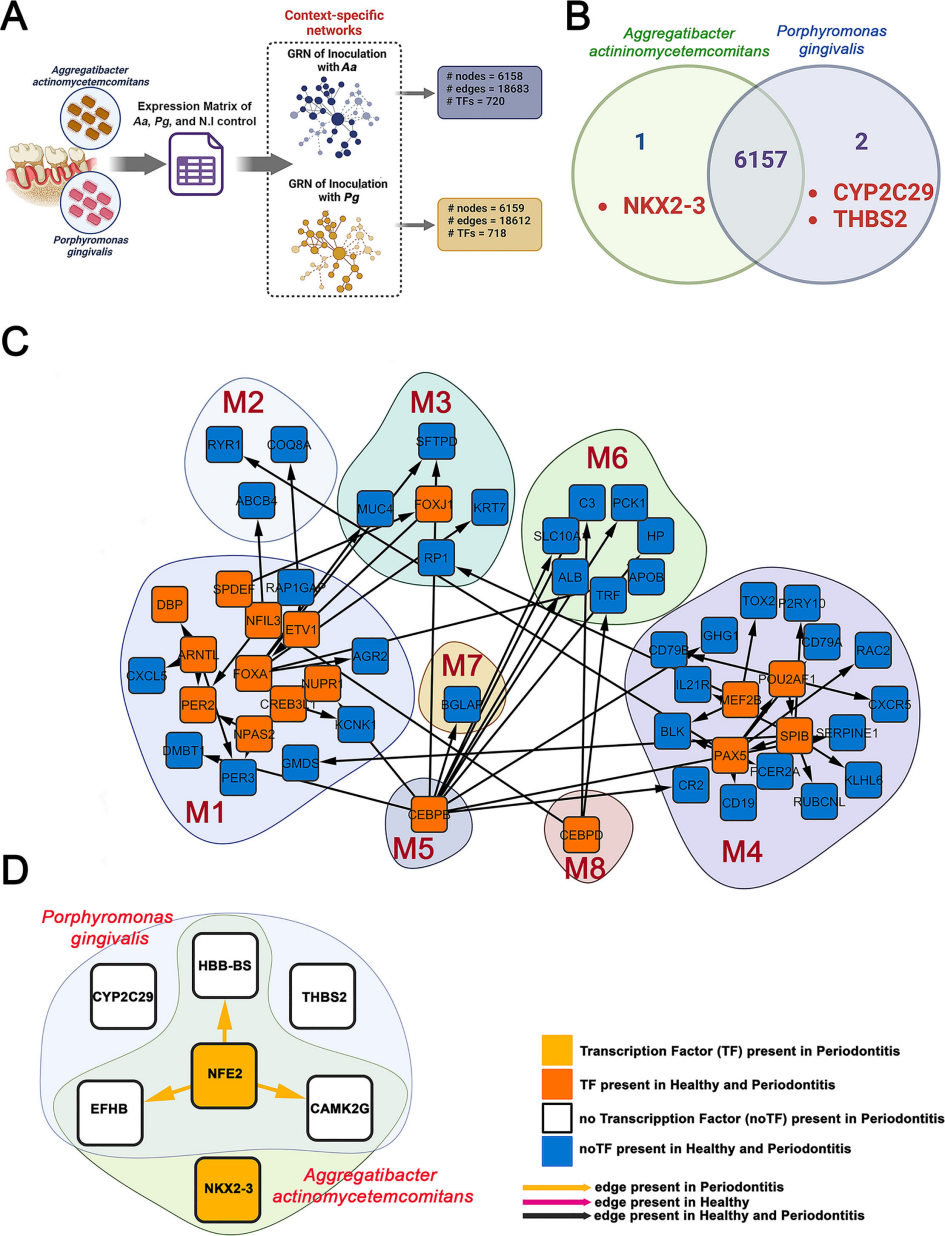
Gene regulatory network analysis reveals shared and distinct transcriptional control mechanisms in *Aa*- and *Pg*-induced periodontitis. (**A**) Workflow schematic illustrating the generation of context-specific regulatory networks from oral mucosal transcriptomes of mice inoculated with *Aa* or *Pg*. (**B**) Venn diagram displaying the overlap of regulatory network nodes between *Aa* and *Pg* GRNs. Red text highlights pathogen-specific nodes (NKX2-3 in *Aa*; CYP2C9 and THBS2 in *Pg*). (**C**) Modular organization of the gene regulatory network grouped by co-expression modules (M1-M8). Nodes represent genes, and edge colors indicate regulatory relationships. (**D**) Differential network analysis highlighting nodes exclusive to *Aa* (green) and *Pg* (light blue) induced periodontitis. Shared nodes are shown in the overlapping region. Bottom right: Network visualization legend describing node shapes, colors, and edge types used in panels C and D.

There is evidence that diseases arise not from alterations in a single gene but from disruptions within the complex networks connecting multiple tissues (30). Therefore, network-based approaches enhance our understanding of the mechanisms underlying human diseases. By employing these methodologies, we can elucidate specific regulatory interactions and pinpoint the genes encoding TFs implicated in various cellular functions associated with health and disease (31–33). Recognizing the significance of TFs in various diseases, including periodontitis (19, 32, 33), we utilized our transcriptomic data to construct GRNs. We then performed differential network analysis to identify the full repertoire of TFs regulating gene transcription levels, revealing the key interactions underlying the pathological phenotype shown in Fig. 1B and C. First, we construct context-specific GRNs using the normalized expression count matrix derived from the expression analysis and the TFs’ interactions with their high-confidence targets, in accordance with previously published protocols (19, 34). Two extensive networks covering health and disease contexts comprise 6,158 nodes, including 720 TFs and 18,683 edges, when *Aa* induces periodontitis (Fig. 6A and Fig. S2A), and a second network comprises 6,159 nodes, 718 TFs, and 18,612 connections when *Pg* causes periodontitis (Fig. 6A and Fig. S2B). Notably, these networks share 6,157 genes, where the gene encoding the TF NK2 Homeobox 3 (*NKX2-3*) is exclusively present in the *Aa-*induced *GRN*, while the protein-coding (non-TF) genes Cytochrome P450 Family 2 Subfamily C Member 9 (*CYP2C9*) and Thrombospondin 2 (*THB2S*) are only found in the GRN of *Pg* (Fig. 6B). The main finding evidenced by these figures (6A, 6B, and S2A and B, Supporting Information) indicates that although these pathogens explore different pathways to develop their pathological activities (Fig. 2–5), the same TFs regulate them at the transcriptional level. A subnetwork was then created from these networks, including only the nodes belonging to the eight co-expression modules identified using CEMiTool. This subnetwork comprised eight groups and their corresponding regulatory interactions, containing 54 nodes, 17 TFs, and 53 edges. (Fig. 6C). In the subnetwork examined, M4 is the largest node cluster and the hub of the host-immune response. This module comprises 19 nodes, of which 4 correspond to TFs. Notably, the TFs identified as Myocyte Enhancer Factor 2B (*MEF2B*), Paired Box 5 (*PAX5*), Spi-B Transcription Factor (*SPIB*), and POU Class 2 Homeobox Associating Factor 1 (*POU2AF*) are present in both healthy and disease conditions, with no DEGs or TFs exclusively associated with periodontitis. These transcription factors interact with other modules, such as M1 (*GMDS*), M2 (*RYRI*), and M3 (*RP1*), and regulate genes within the same M4. The second-most-represented module is M1, with 17 nodes. However, this module stands out for housing the largest number of TFs. The 10 TFs identified in this module perform regulatory activities within M1 and show regulatory interactions with modules M2, M3, and M6. In M1, the TF with the most regulatory interactions is Forkhead Box A1 (*FOXA1*). It regulates several genes: *AGR2* in module M1; *TRF* in module M6; and *KRT7*, *MUC4*, and *SFTPD* in module M3. Additionally, *FOXA1* is regulated by Forkhead Box J1 (*FOXJ1*)-TF (M3), which in turn is regulated by the SAM Pointed Domain Containing ETS Transcription Factor (*SPDEF*) of the M1. Another prominent TF in the M1 module is *ARNTL*, also known as basic helix-loop-helix ARNT-like (*1BMAL1*). This factor regulates *DBP* and *PER2*-TFs*,* as well as the *CXCL5-* and *PER3-*encoding genes within the same M1 module. Furthermore, no regulatory interactions between *ARNTL* and other modules were observed. The TFs CCAAT Enhancer Binding Protein Beta (*CEBPB*) and CCAAT Enhancer Binding Protein Delta (*CEBPD*), the sole representatives of modules M5 and M8, play crucial roles in various biological functions. For example, *CEBPB* promotes osteoblast differentiation and osteoclastogenesis regulation (35), contributes to granuloma formation, and activates CD4^+^ T cells and macrophages during inflammatory immune responses (36). Numerous genes, such as *DMBT1* and *NUPR1* in M1, *SFTPD* in M3, *BGLAP* in M7, and *CR2*, *SERPINE1*, and *CHG1* in M4, are modulated by *CEPB* in our regulatory network. Additionally, it regulates *HP*, *PCK1*, *ALB*, and *SLC10A* in M6, highlighting its extensive regulatory capacity. For its part, *CEBPD* is related to regulating the inflammatory immune response triggered by infectious diseases and the IL-17 family signaling pathways (37, 38). This TF regulates the *NFIL3* genes in M1 and *C3* and *TRF* in M6 within our regulatory network. Lastly, M2, M6, and M7 do not possess TFs. Therefore, their genes are regulated by TFs from other modules, as previously described (Fig. 6C). To analyze our data further, we focused exclusively on nodes associated with *Aa*- or *Pg*-induced periodontitis, highlighted in green and light blue, respectively, in the subnetwork shown in Fig. 6D. These genes were selected because they exhibited the highest variation in their local proximity, as measured by the LoTo F1 metric (F1 < 0.99). This metric evaluates whether the neighborhood of a gene in a GRN changes when comparing two networks under different contexts, in this case, *Aa-* or *Pg*-induced periodontitis. Lower F1 values indicate more significant alterations in the GRNs. Our analysis identified a subnetwork comprising five genes, two of which are TFs, following inoculation with *Aa*. Among these TFs, NK2 Homeobox 3 (*NKX2-3*) is notable as it appears solely in the *Aa*-associated subnetwork. This gene is linked to inflammatory bowel disease 1 and thyroid malformations. Likewise, its paralog, *NKX2-5*, plays a vital role in cardiac conduction during development. The transcription factor Nuclear Factor Erythroid 2 (*NFE2*) also regulates the genes *HBB-BS*, *EFHB*, and *CAMK2G*, which are included in both subnetworks (*Aa* and *Pg*). In contrast, the genes *CYP2C29* and *THBS2*, found exclusively in the *Pg* subnetwork, do not show any regulatory interactions with the TFs identified in Fig. 6D. This finding indicates a similarity between gene regulation and central organization between the two analyzed subnetworks.

### Analysis and identification of master regulator genes in inoculation-induced experimental periodontitis

**Fig 7:**
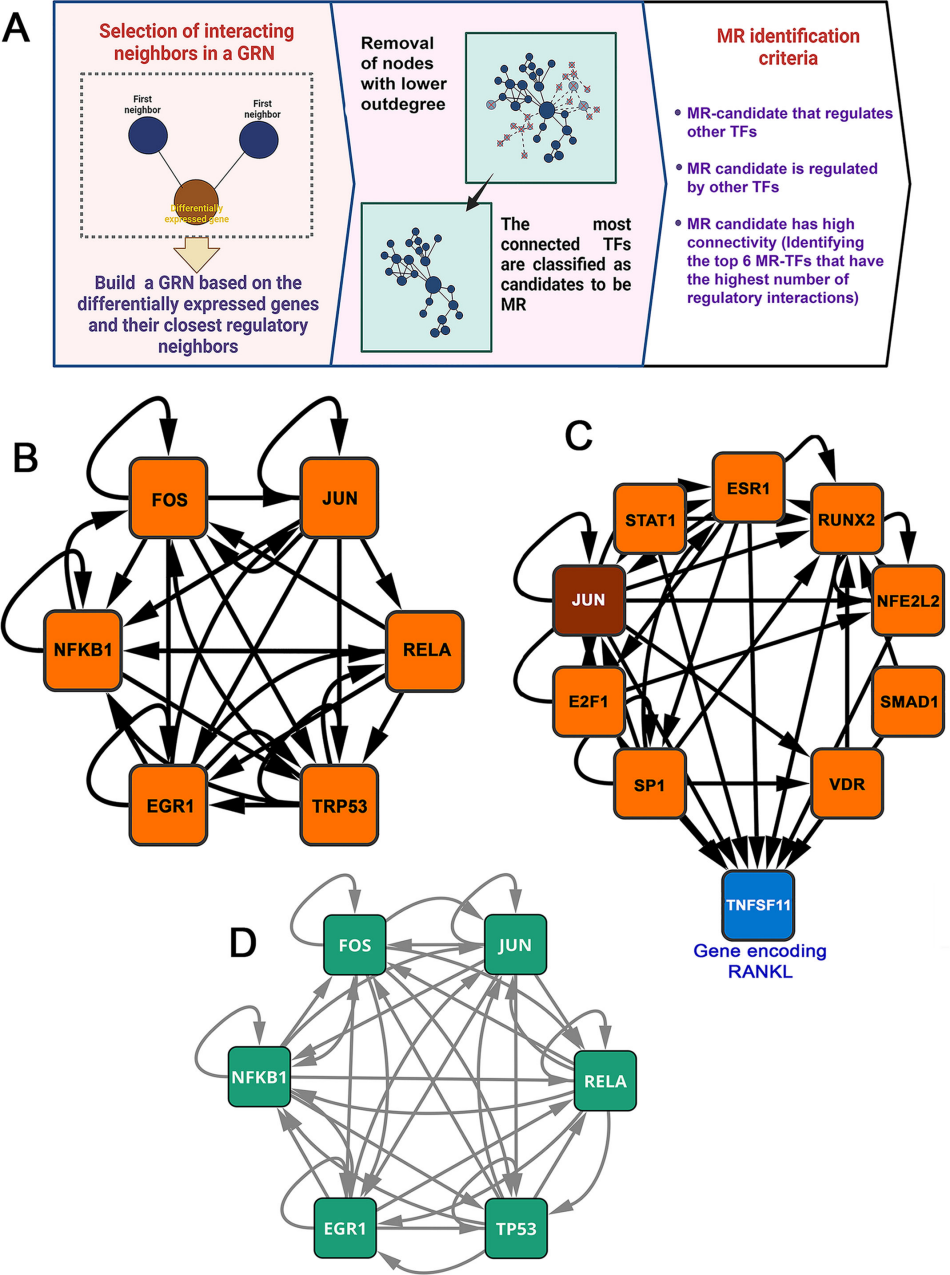
Master regulator transcription factors orchestrate the host response in experimental periodontitis. (**A**) Schematic workflow for identifying MR-TFs in *aa*- and *Pg*-induced periodontitis. (**B**) Core regulatory subnetwork showing interactions among the six identified MR-TFs. (**C**) Regulatory network controlling RANKL (*TNFSF11*) expression. The dark red square indicates differential expression in periodontitis. JUN (highlighted) is the only master regulator in networks **B** and **C**. (**D**) Structure of the central regulatory subnetwork mapped onto the human reference network (TFlink). Note the high degree of topological conservation with respect to the original murine network shown in panel B.

A master regulator (MR) is a transcription factor that operates at a high hierarchical level within a GRN. It is characterized by multiple regulatory interactions that allow it to coordinate a wide range of functionally related target genes. This important position enables the MR to initiate, maintain, or reprogram a specific cellular state or phenotype, such that its disruption causes profound changes in the network’s topology, architecture, and overall function (39, 40). Following established protocols (19, 41), we used the DEGs (*Aa* and *Pg*) identified in our transcriptomic analyses as “seed genes” to build the subnetwork. For each seed gene, we included its first neighbors—genes that are directly connected through regulatory interactions or high-confidence co-expression. We then expanded the network to include second neighbors, defined as genes indirectly linked to the seed gene via first neighbors. The resulting directed network was filtered by removing nodes with an indegree and outdegree (regulatory interactions) of ≤4, ensuring that only the most informative regulatory components were retained. After several iterations of eliminating nodes with low connectivity, we obtained a compact and highly clustered subnetwork of TFs consisting of nodes with indegrees and outdegrees >3 (Fig. 7A). Six MR-TFs associated with experimental periodontitis induced by inoculation were identified from the DGEs of our transcriptomics data. These are Transformation-Related Protein 53 (*TRP53*), Nuclear Factor Kappa B Subunit 1 (*NFκB1*), Jun Proto-Oncogene, AP-1 Transcription Factor Subunit (J*UN*), RELA Proto-Oncogene, *NFκB* Subunit (*RELA*), Early Growth Response 1 (*EGR1*), and Fos Proto-Oncogene, AP-1 Transcription Factor Subunit (*FOS*), forming a subnetwork with 28 connections (Fig. 7B). Within this context, *TRP53* emerges as the main regulator, with the highest number of regulatory interactions with 11 edges. It is followed closely by *EGR1* and *FOS*, each with 10 interactions; *NFKB1* and *JUN* have 9 interactions; and *RELA* has 7 interactions. The analysis of global networks (Fig. S2A and B) revealed a similar trend, identifying *TRP53* as the MR with the highest number of genes under its regulation. This TF revealed 431 nodes and 454 regulatory interactions, comprising 59 indegree and 395 outdegree connections. *RELA* was found to modulate 280 nodes, with 284 interactions, comprising 12 indegree and 272 outdegree connections. Similarly, *EGR1* was associated with 203 nodes and 212 interactions, including 27 indegree and 185 outdegree connections within both global networks (Table 1). Conversely, *NFκB1*, *JUN*, and *FOS* displayed minimal differences in the number and type of connections analyzed in the *Aa*-GRN and *Pg*-GRN. Nonetheless, they regulate the same number of nodes in both global networks. After *TRP53*, *NFkB1*-MR is the second regulator with the most genes, covering 378 nodes with 393 interactions in the *Aa* network and 392 in the *Pg* network. *JUN*, for its part, regulates 330 nodes through 341 edges in the *Aa* network and 342 in the *Pg* network. Finally, FOS regulates 192 nodes, with 201 edges in the *Aa* network and 202 in the *Pg* network (see Table 1 for more details). Additionally, we constructed a subnetwork integrating DEGs with those regulating the expression of Receptor Activator of Nuclear Factor-κB ligand (RANKL), which encodes a key protein involved in osteoclastogenesis and subsequent pathological alveolar bone resorption during periodontitis (42, 43). This approach enabled us to identify 9 MR-TFs and 35 specific connections. Among the genes analyzed, *JUN* is notable for its differential expression and for being the only MR present in both subnetworks (Fig. 7B and C). This finding suggests that JUN may be crucial in regulating signaling pathways involved in inflammation and bone metabolism, which are key determinants of periodontitis progression.

**TABLE 1:** The table presents the number of regulatory interactions, including indegree and outdegree, associated with the MR-TFs identified in this study, as well as the top 100 genes they control within the transcriptional regulatory networks related to periodontitis induced by *Aa* and *Pg*

Symbol	Description	Regulatory interactions						Top 100
		*Aa*			*Pg*			
		Indegree	Outdegree	Total	Indegree	Outdegree	Total	
*FOS*	Fos proto-oncogene, AP-1 transcription factor subunit	55	146	201	56	146	202	*IL2 ARNT LRIG2 ATF1 MMP2 CSTAT3 CREB5 NFB2L2 TPSAB1 PDPN FIGF VDR NFATC1 RUNX2 LOXL4 SRXN1 CXCL15 CREB3L2 NOS1 STAT3 ETS2 EP300 GREB1 PGF NEFL BATF3 NTS SRC CSTAT2 BCL2L11 FAM89B MITF FASN MYC BDNF DCSTAMP VEGFC BCL2L1 MELTF CDH1 EGR1 PGR BMP2 SPRR3 TNFAIP6 STAT1 NAT2 STAT6 NACC1 STFA2 EZR GRIK2 MMP1B SP1 CCND1 IL1B VEGFD KCNIP3 MMP10 TRP53 KRT18 ELK1 ERCC4 STFA1 GSTP1 SRF CREBBP RELA NPPA IL1A FOSB PLAUR ETS1 VEGFA GATA3 MAF ACP5 FOSL1 TSLP SOCS3 ESR1 CRH CLU RB1 GTF2I CREB3L1 IL12B CD69 NOS2 HSPH1 PPARD RBPJ NAT1 PDHA1 TFF2 CSF3 FAS HDAC3 ST1M1 CREM*
*JUN*	JUN proto-oncogene, AP1 transcription factor subunit	33	308	341	34	308	342	*CCL2 DCSTAMP COL10A1 AANAT BATF3 SEPI1 GAST PDK1 SMURF1 KLK1B4 TBP RELA EMILIN1 PPP1R3B PEA15A PLAT NCK2 CSTA2 NACC1 LTF MMP7 PPARD COL7A1 GZMB ABCB1B TWIST1 MEF2A UCP2 TCF7 STNM1 LOC640611 KLF4 ANGPT2 STAR KRT16 RB1 ALDH3A1 TXN1 STFA2 C1QTNF3 DBH ACP5 IL5RA HEY1 CXCL15 ITGAX TRP73 PTGS2 KRT18 BCL3 IFNB1 IFNE SLC2A1 SRXN1 LEF1 MMP10 MMP9 EGR1 HDAC1 NTS TRP53-PS NPY SPP1 REN1 VCAM1 HFE CCND2 MT3 APOC3 NUDT1 CSTA3 NAT1 SLC38A1 ELAVL1 MYB NQO1 CCND1 CD14 WEE1 COL3A1 ETS1 PENK ECM1 CD5 TTR MYOD1 HDAC3 CYP2J9 SFTPB PLAU BDKRB1 GAD1 NFATC1 PRDM1 IL24 RUNX2 NAT2 ATF2IGK AR NGF SRT1*
*RELA*	RELA protooncogene, NF-kB subunit	12	272	284	12	272	284	*CCL2 FN1 CCL11 IFNA1 LTC4S RIPK2 BAX KLK1B4 CEBPG IFNA12 TRAF2 IRF7 CD40 CD80 TRAF4 PPARD THBD CD83 IL17C PIGR ABCB1B TWIST1 TCRG-C1 KDM2A MILL1 MGMT COL1A2 MAP3K8 HIF1A GSTP1 TNFRSF4 PSME2 SLC1A2 CXCL15 SOX9 TRP73 PTGS2 BCL3 INFB1 AMH KLRB1C SOD2 GABRE PARP1 MMP10 CCL4 S100A6 MMP9 SELP SNAI1 EGR1 NOL3 HDAC1 NR4A1 VCAM1 IFNA7 DCST2 GATA3 KLK1B16 AKT1 MYB CCND1 HSD11B2 IFNA13 KLF5 IFNA9 STAT3 CTSB KLK1B9 KLK1 DCST1 HES1 CD38PLAU SHH KLK1B22 EPAS1 TRAF6 IFNA2 IGF2BP2 PTEN ATF2 IGK AR SIRT1 FCGRT CFLAR KLK1B3 RORC KLK1B24 DMP1 PCNA OPTN IL1B PCK2 CDK4 COL2A1 CDKN1B DUOX2*
*EGR1*	Early response growth 1	27	185	212	27	185	212	*CCL2 FN1 TKI KCNH8 BAX RELA POR FGF2 ID3 CRABP2 FASNIFNGR1 GALK1 MAPK14 ABCB1B TWIST1 LHB MAPK1 STMN1 FLT1 FLT3 LMTK2 COL1A2 UBE2S HIFIA CDKN1C TRP73 PTGS2 CYR61 NDRG1 MMP9 ALOX5 SOCS1 ELK4 NOL3 NR4A1 CD9 TUBB4B SPP1 ANGPTL4 NAB2 FGFR3 CCND3 CDH13 FOXO1 SLC1A4 RBL2 MYB CCND1 HSD11B2 KLF5 STAT3 MAP1LC3B PLAU GAD1 ELK1 PTEN SLC9A3 AR STAT1 CTSL AKR1B3 CREBBP IL1B TPO COL2A1 ABCA2 GDF15 FASL SERPINE1 GDNF NGB CHRNA7 IGF2 CD19 FCER2A EPX DMTF1 PTP4A1 EGFR ACE IGF1R ATF3 TBXA2R RCAN1 PTGES2 ITGA7 LTB CDK5R1 IMPDH2 PNMT TCF4 JUN EAPP IFNGR2 PTPRZ1 TGFB1 IFNG WT1*
*NFκB1*	Nuclear factor kappa B subunit 1	41	352	393	40	352	392	*CCL2 DCSTAMP FN1 CCL11 IL15 ZFP384 PTGER2 RIPK2 RELA JUNB CEBPG TRAF2 IRF7 CD40 CD80 TRAF4 THBD CD83 NOD2 IL17C PIGR MAPK14 ABCB1B TWIST1 FOXP3 TCRG-C1 KDM2A ADORA1 MILLI1 COL1A2 HIF1A GSTP1 TNFRSF4 ALDH3A1 PSME2 CFB SLC1A2 ACP5 CXCL15 SOX9 PTGS2 BCL3 IFNB1 AMH SLC2A1 SOD2 LEF1 NOTCH1 PARP1 MMP10 CCL4 MMP9 EIF2AK2 SELP ALOX5 EGR1 NOS1 CRMP1 NOL3 ITGAM TRP53-PS BTK ADORA2B UGCG VCAM1 CCND2 CENPJ GCLM IL1RN DCST2 APOC3 GATA3 GLRX NUDT1 KLK1B16 AKT1 MYB NQO1 CCND1 HSD11B2 IRAK2 KLF5 MMU-MIR 146A-5P ETS1 CTSB KLK1B9 KLK1 A2M THRB DCST1 HES1 PIRB SERPINB1A CD38 PLAU SHH GAD1*
*TRP53*	Transformation-related protein 53	59	395	454	59	395	454	*PDGFRB TRP53INP1 BID BAK1 SMURF1 DAGLB FGF2 SIAH2 APC LTF TWISTI MAPK1 FOXP3 STMN1 KLF4 RB1 RPS6KA1 GSN GSTP1 HIF1A CDKN1C CX3CL1 MRPL27 TRP73 S100A4 KAT2B RAP2B NOTCH1 HIPK2 MYCN CSNK2A2 NUPRI EGR1 CCNG2 ELF4 DUSP4 SPP1 HIPK1 CDH13 CASP7 SOX6 ANKRD1 RNF144B KLK1B16 PLK2 TEAD2 NROB2 TYMS KLK1B9 PRNP STAT3 FRMD5 MYOD1 PROX1 PIK3CA ASCC3 PRDM1 OGG1 BNIP3L SIRT1 INSR TGFA KLK1B24 STAT1 WWP1 FKBP3 PLAGL1 SERTAD1 COL2A1 ADGRB2 CDKN1B CTSD LIG1 ID2 BTG2 CABLES2 CASP6 OLFR17 TYR LRDD CCNG1 PRKAB1 PSME3 ATM NANOG AFP NFIC EXPI CKAP2 ESRRA BBC3 EZH2 KIT CCNB1 PYCARD HRAS PCBP2 KLK1B8 BDKRB2 LITAF*

To assess the degree of evolutionary conservation and translational relevance of this regulatory program, we mapped the murine network onto the TF-link reference human regulatory network using orthologous assignment (44). Notably, the six transcription factors maintained similar topological characteristics in the human network, remaining central hub nodes (Fig. 7D). This finding demonstrates the structural conservation of the inflammatory circuit between the two species. Furthermore, after incorporating genes related to bone metabolism (Fig. 7C) and projecting this expanded network onto the human TF-link, we identified the same hierarchical structure and regulatory stability among the nine transcription factors (Fig. S3A). Overall, these results suggest that the experimental periodontitis model induced by *Aa* or *Pg* inoculation recapitulates key regulatory programs observed in humans, thereby supporting the biological validity and translational relevance of our study.

Altogether, these findings indicate that, despite phylogenetic differences and the distinct virulence repertoires of *Aa* and *Pg*, a common set of MR-TFs regulates transcription during their pathogenesis. These factors coordinate specific regulatory programs that integrate various signaling pathways and biological processes conserved in humans, which may directly contribute to mechanisms leading to periodontal destruction.

## DISCUSSION

Periodontitis is a complex disease whose clinical presentation and progression are influenced by multiple factors (45). Transcriptomic analysis of periodontitis-affected tissues has provided valuable insights into its underlying pathobiological mechanisms (46–48) and molecular characterization of its clinical phenotype (49, 50). In this study, we used an experimental periodontitis model by directly inoculating *Aa* or *Pg* into murine periodontal tissues. Although periodontitis is widely recognized as a dysbiotic disease rather than an infection caused by specific pathogens (5), our transcriptomic analysis revealed substantial gene expression changes associated with each bacterium. Despite differences in pathogenicity strategies and specific virulence factors of *Aa* and *Pg*, both converge on a highly conserved transcriptional regulatory framework articulated by six MRs, whose structure is preserved in humans.

Only 22 DEGs were shared between *Aa* or *Pg*-induced periodontitis, underscoring pathogen-specific molecular responses. Functional enrichment analyses revealed that *Aa* primarily activated pathways related to extracellular matrix remodeling, immune responses, and vesicular trafficking, whereas *Pg* predominantly activated pathways related to cell division and metabolic processes. Notably, over half of the shared DEGs were linked to humoral immune responses, suggesting that, despite distinct initial pathogenic mechanisms, both bacteria ultimately converge on adaptive immunity-related pathways. Variations in gene co-expression were also evident, with seven of the eight identified co-expression modules displaying distinct expression patterns across pathogens. These differences may explain variations in the progression of periodontitis caused by phylogenetically distinct bacteria. Interestingly, module 4, associated with the immune response, was the only module upregulated in both conditions. This suggests that while the bacteria employ different virulence strategies, they ultimately trigger similar immune-mediated tissue destruction (50–52).

The identification of six MR-TFs (*TRP53*, *NFκB1*, *RELA*, *EGR1*, *FOS*, and *JUN*) represents a significant advancement in understanding the pathogenesis of periodontitis. Among them, *TRP53* emerged as the primary regulator due to its extensive interactions (Table 1), highlighting its critical role in orchestrating the host response to periodontopathogens in the periodontium. *TRP53* encodes p53, a transcription factor that regulates cell cycle arrest, apoptosis, senescence, DNA repair, and metabolic adaptation (53). Beyond its well-known role in tumor suppression, TRP53 has been implicated in chronic inflammation, macrophage polarization, and immune modulation in several diseases, including rheumatoid arthritis, tuberculosis, and sepsis (54–57). Using the ligature-induced periodontitis model, increased infiltration of p53-positive macrophages was observed in periodontitis-affected tissues, whereas p53-deficient mice exhibited more severe disease and higher numbers of pro-inflammatory M1 macrophages (58).

Similarly, *NFκB* plays a pivotal role in inflammatory and adaptive immune responses. Functioning as a homodimer or heterodimer (with subunits such as *RELA/p65*, *RELB*, *NFκB1/p105*, *NFκB1/p50*, and *NFκB2/p52*), *NFκB* regulates cell differentiation, proliferation, immune evasion, and apoptosis (59–61). Given its role in inflammation, it has been extensively studied in numerous inflammatory diseases, including periodontitis (61). Another critical MR-TF, *EGR1*, is involved in cell growth, differentiation, and apoptosis (62). It plays a key role in wound healing, tissue remodeling, and fibrosis and has been linked to ischemic lesions, cancer, atherosclerosis, and cardiovascular disease (62–66). Recent studies indicate that *EGR1* regulates immune gene expression and contributes to an immunosuppressive microenvironment in periodontitis (67). Tissue destruction in periodontitis activates *EGR1*, potentially linking it to the host immune response and disease progression. Additionally, *FOS* and *JUN* heterodimerize to form the Activator Protein-1 (AP-1) complex, a key regulator of immunity, bone metabolism, and inflammation (68–70). *FOS* is activated by cytokines and stress stimuli and regulates cell proliferation and differentiation. It also plays an essential role in neuroplasticity and carcinogenesis. In osteoimmunology, *FOS* mediates immune-bone interactions, linking chronic inflammation to alveolar bone loss (68). *JUN*, in turn, regulates oxidative stress responses, cytokine production, and fibroblast activation, promoting extracellular matrix remodeling and re-epithelialization (71–75). Importantly, *JUN* was the only MR-TF present in both the global regulatory network and the RANKL-specific network, suggesting a potential mechanistic link between immune responses, tissue remodeling, and pathological bone loss (76–79).

In the context of this study, the murine model of pathogen-inoculation-induced periodontitis is presented as an efficient experimental tool for unraveling molecular and transcriptomic mechanisms underlying both microbial virulence and host immune activation. However, despite its usefulness, it is necessary to acknowledge certain limitations inherent in its ability to reproduce human periodontitis. First, the murine model used in this research does not fully replicate the complexity of human periodontitis, which is influenced by host genetic variability, environmental factors, and the interplay of a multispecies dysbiotic biofilm. In this context, this model simplifies the polymicrobial nature of human periodontitis by focusing on a dominant single pathogen. In humans, the disease arises from heterotypic, multispecies microbial communities that form complex consortia capable of enhancing pathogenicity through metabolic cooperation, collective immune evasion, and joint resistance (5, 6). While our model allowed a controlled comparison of *Aa*- or *Pg*-induced periodontitis, enabling analysis of specific virulence characteristics and pathogenicity strategies, it does not account for the potential synergistic effects of multispecies-heterotypic communities that coexist and structure the subgingival biofilm during human periodontal disease. Additionally, direct inoculation initiates an acute, targeted infectious process that does not replicate the gradual ecological transition or the temporal evolution of periodontal damage. Since this study evaluated the transcriptome at a single time point (30 days post-inoculation), the results may not capture the progressive, chronic dynamics that characterize human periodontitis. Future studies incorporating time-course transcriptomic analyses and *in vitro* validation of the identified MR-TFs could provide further insights into the regulatory mechanisms underlying periodontitis. Furthermore, while we employed stringent bioinformatic pipelines to construct GRNs, functional validation through knockdown or overexpression experiments would strengthen the causal link between MR-TFs and disease progression.

Despite these limitations, our findings provide a robust molecular framework for understanding the transcriptional regulation of *Aa* or *Pg*-induced periodontitis, paving the way for future studies on targeted therapeutic interventions. For instance, our findings align with evidence demonstrating that *Aa* and *Pg*, along with their purified virulence factors, stimulate the expression and activation of the 6 MR-TFs, which are involved in inflammation, immunity, and tissue destruction. Both live bacteria and their components (LPS, toxins, and fimbriae) promote immune evasion, osteoclastogenesis, and alveolar bone loss, which are key processes in the progression of periodontitis (16, 80–96). Furthermore, in our previous study using ligature-induced periodontitis, we identified 26 MR-TFs, including the 6 MRs found in the present study (19). The ligature model, widely used due to its ability to mimic microbial dysbiosis and destructive inflammation, has limitations in fully capturing pathogen-specific immune responses (97). However, the presence of *RELA*, *NFKB1*, *JUN*, *FOS*, *TP53*, and *EGR1,* as central nodes in the murine model of ligature-induced periodontitis (Fig. S3B), reveals a robust regulatory core that sustains the periodontal inflammatory response. Although ligature induces inflammation through mechanical damage and dysbiosis, while *Aa* and *Pg* do so through virulence factors, the recurrence of these six MR-TFs indicates that different stimuli converge on a common transcriptional program that coordinates inflammatory, immunological, and osteoimmunological pathways associated with tissue destruction. Complementarily, the presence of a second-order network (light blue nodes in Fig. S3B) in the ligature model suggests activation of processes associated with immunomodulation, metabolic regulation, and tissue remodeling in response to chronic persistent stimulation triggered by the dysbiotic microbiota. The biological relevance of these findings lies in the fact that the identified hierarchical architecture, comprising 26 MRs and a central core of 6 MR-TFs, remains conserved when projected onto human reference networks (Fig. S3C). Although there are differences in the total number of regulatory interactions, 169 in the mouse network (S3B) compared to 619 in the human network (S3C), likely attributable to differences in the density and depth of the information used to construct these reference networks, the conservation of the nodal hierarchy is particularly significant from a translational perspective. This aligns with previous evidence demonstrating that, despite species-specific differences, the transcriptional programs that regulate the immune response and effector cell differentiation exhibit substantial conservation between mouse and human, both in their expression signatures, regulatory circuits, and MRs that govern them (98). This interspecies conservation not only confirms the usefulness of preclinical murine models but also reinforces the functional interpretation of the identified MR-TFs and positions this central regulatory core as a potential strategic axis for the development of therapeutic interventions with direct application in human periodontitis.

Periodontitis significantly impacts public health, contributing to the global burden of chronic diseases and reducing patient quality of life (99–101). This study provides a molecular framework that illustrates how distinct periodontal pathogens induce similar damage through divergent mechanisms yet share common transcriptional regulators. These findings have critical therapeutic implications. While initial antimicrobial strategies should target specific pathogens, modulating MR-TFs, such as *JUN*, *FOS*, *NFκB*, and *TRP53*, could offer broad-spectrum treatment options independent of the bacterial species involved. Additionally, differences in the pathways triggered by the pathogens in this study may explain why patients with periodontitis and other chronic inflammatory conditions respond differently to treatment, potentially aiding the development of more personalized treatment strategies (102–105).

The current study highlights that, despite their evolutionary and phylogenetic differences and unique virulence factors, *A. actinomycetemcomitans* and *P. gingivalis* activate common transcriptional regulators that drive the progression of periodontitis. The identification of shared MRs of transcription underscores the conserved molecular mechanisms underlying periodontal destruction. This discovery opens new avenues for targeted interventions to modulate these regulatory pathways, potentially improving treatment outcomes for periodontitis and related inflammatory diseases.

## MATERIALS AND METHODS

### *A. actinomycetemcomitans* and *P. gingivalis* strains and growth conditions

Periodontal infections were induced using *Aa* ATCC43718 (serotype b) and the encapsulated strain W85 of *Pg* (serotype K1). The strains were cultured at 37°C under capnophilic or anaerobic conditions, as described by Monasterio et al. (106) and (107), respectively. Growth curves were obtained following the methods outlined by Vernal et al. (108). To ensure a consistent quantity of bacteria with their full antigenic potential, samples were collected during the exponential growth phase for use in periodontal infections.

### Animals, experimental periodontitis, and tissue sampling

The mice were personally acquired (purchased) and transported by the corresponding author (C.C.) from the Central Animal Facility of the Faculty of Medicine at the Universidad de Chile (Santiago, Chile), in strict compliance with all established biosafety protocols. The experimental groups comprised 6- to 8-week-old wild-type BALB/c mice (five animals per group). These were housed in separate cages and maintained under pathogen-free conditions in a controlled environment with a 12:12 h light-dark cycle, a temperature of 24 ± 0.5°C, 40–70% relative humidity, and air renewal. Throughout the study, the mice were provided with sterile standard chow and water *ad libitum*. The study received approval from the Institutional Animal Care and Use Committee (Protocol code: BIOPUCV-BA 686-2023) and adhered to the ARRIVE guidelines (109). All experiments adhered to the recommendations of the American Veterinary Medical Association (AVMA) (110). The periodontitis was generated, as described by Monasterio et al. (107). Periodontal infections were induced by directly microinjecting *A. actinomycetemcomitans* or *P. gingivalis*. Each BALB/c mouse received an injection of 10 μL of a carefully prepared bacterial inoculum in phosphate-buffered saline (PBS), mixed with 2% carboxymethylcellulose to enhance retention at the injection site. The injections were administered bilaterally into the palatal interproximal gingiva between the first and second molars, using a 26-gauge syringe (Hamilton Company, Reno, NV, USA) and targeting the area closer to the second molar. The procedure was repeated at 48 and 96 h. A group of uninfected animals served as a negative control. Thirty days after the final inoculation, the mice were euthanized with a single overdose of ketamine and xylazine. The complete palatal mucosa was then carefully removed for RNA sequencing analysis. Additionally, the remaining maxillary alveolar bone was utilized to conduct analyses of alveolar bone loss using micro-CT.

### Analysis of alveolar bone loss

Bone resorption was assessed using micro-CT, as previously reported (19). Hemi-maxillae were dissected to remove soft tissues, immersed in a 2.5% sodium hypochlorite solution for 12 h, and washed with 70% ethanol (EtOH), followed by sequential washes with 90% and 100% EtOH for 24 h. The samples were scanned using SkyScan 1272 micro-CT equipment (Bruker, Belgium) at 80 kV, 125 mA, with a rotation step of 0.3° over 360° around the vertical axis and a voxel size of 9 µm. 3D digitized images were generated using NRecom reconstruction software v.1.6.9 (Bruker, Belgium). The images were re-oriented in space using DataViewer software v.1.4.4 (Bruker, Belgium) to standardize the position. Finally, a region of interest (ROI) was defined in the transverse plane using CTan software v.2.2.10 (Bruker, Belgium). The mesial side of the first molar, the distal side of the third molar, and the area between the cemento-enamel junction and the alveolar bone crest were used as reference points for ROI creation and subsequent analysis.

### Total RNA extraction

Total RNA was extracted from complete palatal mucosa using a combination of the TRIzol protocol (#15596026, Invitrogen) and the PureLink RNA Mini Kit (#12183025, Invitrogen). The samples were homogenized with the TissueLyser II (QIAGEN). The total RNA concentration, quality, and integrity were evaluated using the Infinite 200 PRO NanoQuant (Tecan, Männedorf) and the Bioanalyzer (Agilent Technologies, USA), respectively. Only RNA samples with an RNA integrity number (RIN) ≥ 7 were included for further analysis.

### RNA library preparation and sequencing

As recently reported by Vicencio et al. (19), Illumina sequencing was performed at Genoma Mayor, Universidad Mayor, Chile, with some minor modifications. Total RNA was extracted and treated with DNase I to prevent contamination with genomic DNA. The Quant-iT RiboGreen RNA Assay Kit (Life Technologies) was used to measure RNA concentration, and an RNA 6000 pico chip on the Bioanalyzer 2100 (Agilent Technologies) was used to assess RNA integrity. RNA libraries were then constructed using the Illumina TruSeq Stranded mRNA LT Sample Preparation Kit (Low-Throughput Protocol) according to the manufacturer’s guidelines. The Ribo-Zero rRNA Removal Kit (human/mouse/rat) was used to deplete rRNAs from 500 ng of total RNA. The remaining RNA was fragmented using high-temperature divalent cations and converted to cDNA through reverse transcription during first-strand synthesis. The second-strand synthesis then followed, generating double-stranded DNA, which was subsequently end-repaired and adenylated at the 3′ ends. Universal adapters were ligated to the cDNA fragments, and PCR was performed to generate the final sequencing library. The library was validated using a DNA 1000 chip on an Agilent Technologies 2100 Bioanalyzer, quantified by qPCR, pooled at equal concentrations, and sequenced on an Illumina HiSeq with 100 cycles of paired-end sequencing.

### RNA-Seq data analysis

A quality control check was performed using FastQC, followed by automatic removal of detected adapter sequences using Fastp. Clean reads were then mapped to the mouse genome (GRCm39) using Hisat2 (111), and read summarization was performed with featureCounts (112) utilizing GENCODE vM27 for gene annotation (113). Differential expression analysis was performed with the DESeq2 package in R, applying a log_2_ fold-change threshold of ±1 and an adjusted *P*-value (p.adj) of ≤ 0.05. The normalized read count matrix generated by DESeq2 was then extracted for further analysis. For the analysis of co-expression modules, the R package CEMiTool (28), along with the DESeq2-normalized RNA-seq expression matrix, was used to identify co-expression modules and assess associated pathways. Additionally, we constructed a protein interaction network from STRING version 11.5 (114), focusing on interactions with a combined score ≥ 0.7. This network was then employed to identify co-occurrence modules using CEMiTool, as in the approach used for co-expression data. A GMT file of GO terms from Msigdb (29) was incorporated into the CEMiTool analysis to evaluate the overrepresentation of functional terms within each module, as previously described (19).

### Transcriptomics data-based GRN construction

GRNs were constructed following the previously described methodology (19). First, a reference GRN composed exclusively of genes encoding high-confidence transcription factors was developed. Data from the Dorothea (110), TRRUST (111), and RegNetwork (112) databases were integrated and merged. Subsequently, this reference network was filtered to generate context-specific networks based on the normalized counts obtained from each RNA-seq sample (19, 115). The filtering process was carried out as follows: (i) Regulatory interactions specific to each context were retained only if the transcription factors involved and their target genes presented an expression level greater than 0 in at least one replicate. (ii) In addition, the average of the normalized read counts of the genes was required to be greater than 10. Regulations that did not meet these criteria were discarded, thus obtaining regulatory networks adapted to each context. GRNs specific to the health and experimental periodontitis contexts were built and compared using the LoTo tool to identify network elements whose local topologies differ between the two conditions (116). This determines whether network motifs are present in each context-specific network and creates a visualization file compatible with Cytoscape (117). In addition, it assigns a color code that enables highlighting nodes and edges present in both networks or in one network only. From the LoTo-generated network, a meticulous process was undertaken to select genes present in only one of the two contexts. These genes were examined using the modules previously assigned by CEMiTool, enabling identification of relevant genes in the context of health or inoculation-induced periodontitis.

### Identification of master regulators of inoculation-induced experimental periodontitis

MR-TFs are clusters of highly interconnected TFs that physically interact and are strategically positioned within the network to regulate effector genes that shape the desired phenotype (39). MR-TFs were identified using a seed-gene-based approach. We initially selected seed genes from modules closely associated with periodontitis pathogenesis (M1, M2, M4, and M8), as well as the RANKL gene. Next, we identified the regulators of these seed genes and their regulators to define the MR-TFs. A filtering process was implemented to refine the TFs subnetwork. During this stage, nodes with indegree and outdegree ≤4 were removed to focus the analysis on the most relevant TFs with the highest connectivity within the regulatory network. The MR-TFs subnetwork refinement process was performed iteratively, calculating the indegree and outdegree after removing nodes with low connectivity. This procedure was repeated until a highly clustered subnetwork was obtained, composed solely of nodes with indegree and outdegree >3. The edges of the resulting subnetwork were evaluated and removed based on the absence of documented interactions between TF pairs in the STRING database (109). After this filtering, only the interactions backed by computational or experimental data were retained. TFs that did not meet the predetermined indegree and outdegree criteria were eliminated, yielding a final subnetwork of highly connected TFs relevant to gene-regulation analyses in the context of interest.

### Orthology mapping and human network projection

The initial list of transcriptomically identified MR-TFs was cross-validated against three curated murine regulatory networks: DoRothEA, TRRUST, and RegNetwork. For each transcription factor, its presence in these networks was verified, and both the supporting experimental evidence and the number of associated target genes were documented. To evaluate the evolutionary conservation of these regulatory programs, the consolidated mouse network was projected onto its human counterpart using gene-to-gene orthologous mapping via Ensembl BioMart (118) to identify high-confidence 1:1 orthologs. To assess whether the regulatory relationships identified in the mouse networks were maintained in humans, we then used the human reference network from the TFLink database, which currently includes 19,600 nodes (genes) and 6,739,357 experimentally supported interactions. The orthologous genes present in the murine networks (Fig. 7B and C; Fig. S3B) were queried in the TFLink network, and all regulatory interactions among them were extracted. This procedure enabled reconstruction of three human subnetworks equivalent to the mouse networks (Fig. 7; Fig. S3A and C), preserving interaction directionality and regulatory type when parallel evidence was available in TFLink. Structural comparisons of the resulting networks and evaluations of functional conservation of the MR-TFs were subsequently performed in Cytoscape for visualization and topological analysis.

## Data Availability

All sequence data are accessed through BioProject accession number 1226904.

## SUPPLEMENTAL MATERIAL

Figure S1 spectrum.01678-25-s0001.tif Co-expression modules.

Figure S2 spectrum.01678-25-s0002.tif Context-specific networks.

Figure S3 spectrum.01678-25-s0003.tif Regulatory reference networks.

Supplemental legends spectrum.01678-25-s0004.docx Descriptive legends for Fig. S1 to S3.

ASM does not own the copyrights to Supplemental Material that may be linked to, or accessed through, an article. The authors have granted ASM a non-exclusive, world-wide license to publish the Supplemental Material files. Please contact the corresponding author directly for reuse.
