# Supplementary figures and images for: Transcription-based comparison of Aggregatibacter actinomycetemcomitans or Porphyromonas gingivalis-induced experimental periodontitis
Source: Microbiol Spectr. 2026 Jan 8;14(2):e01678-25. doi: 10.1128/spectrum.01678-25 (PMC12889031; doi:10.1128/spectrum.01678-25)

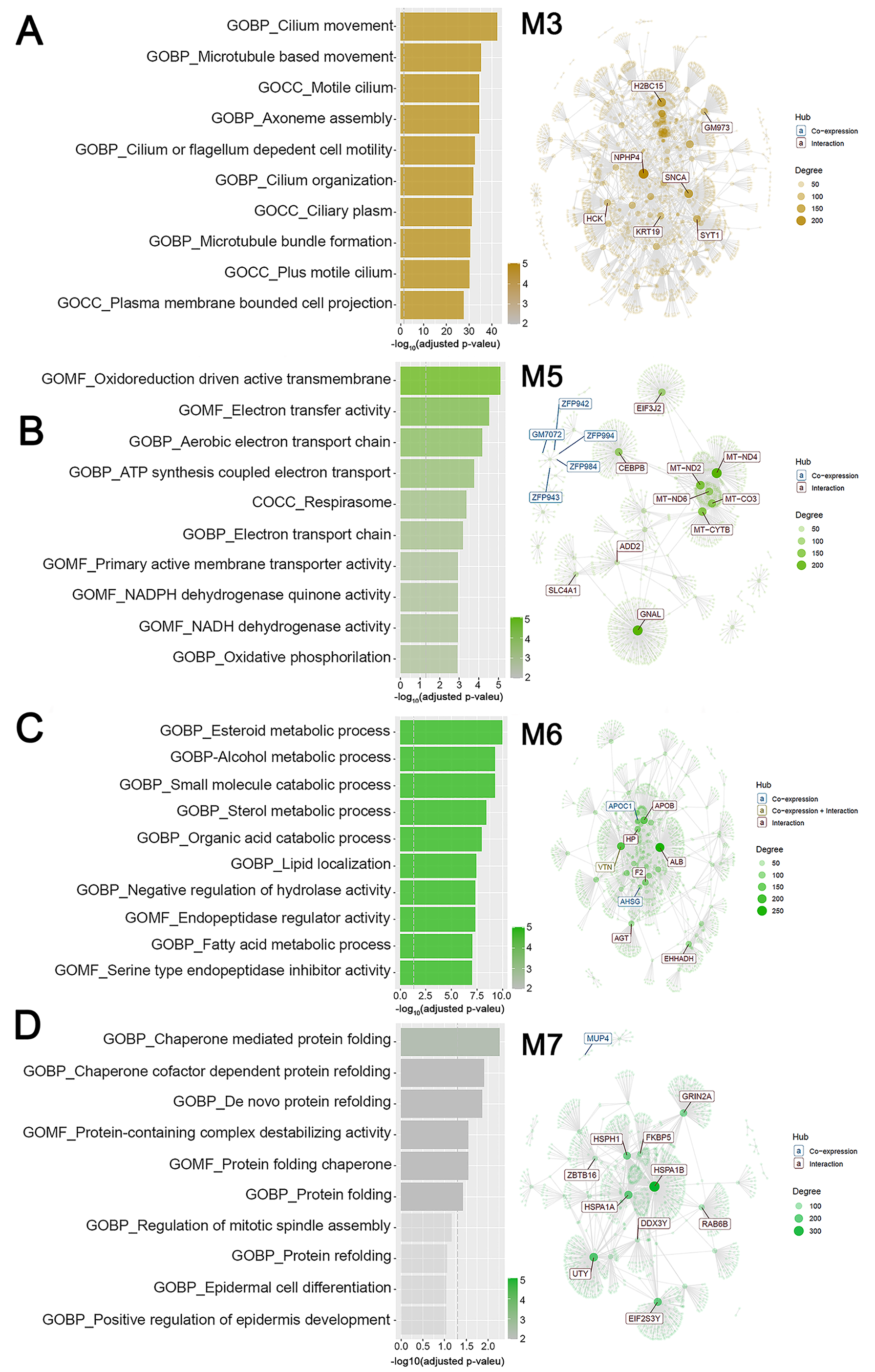

Supplement: Figure S1 — Co-expression modules. [file spectrum.01678-25-s0001.tif]

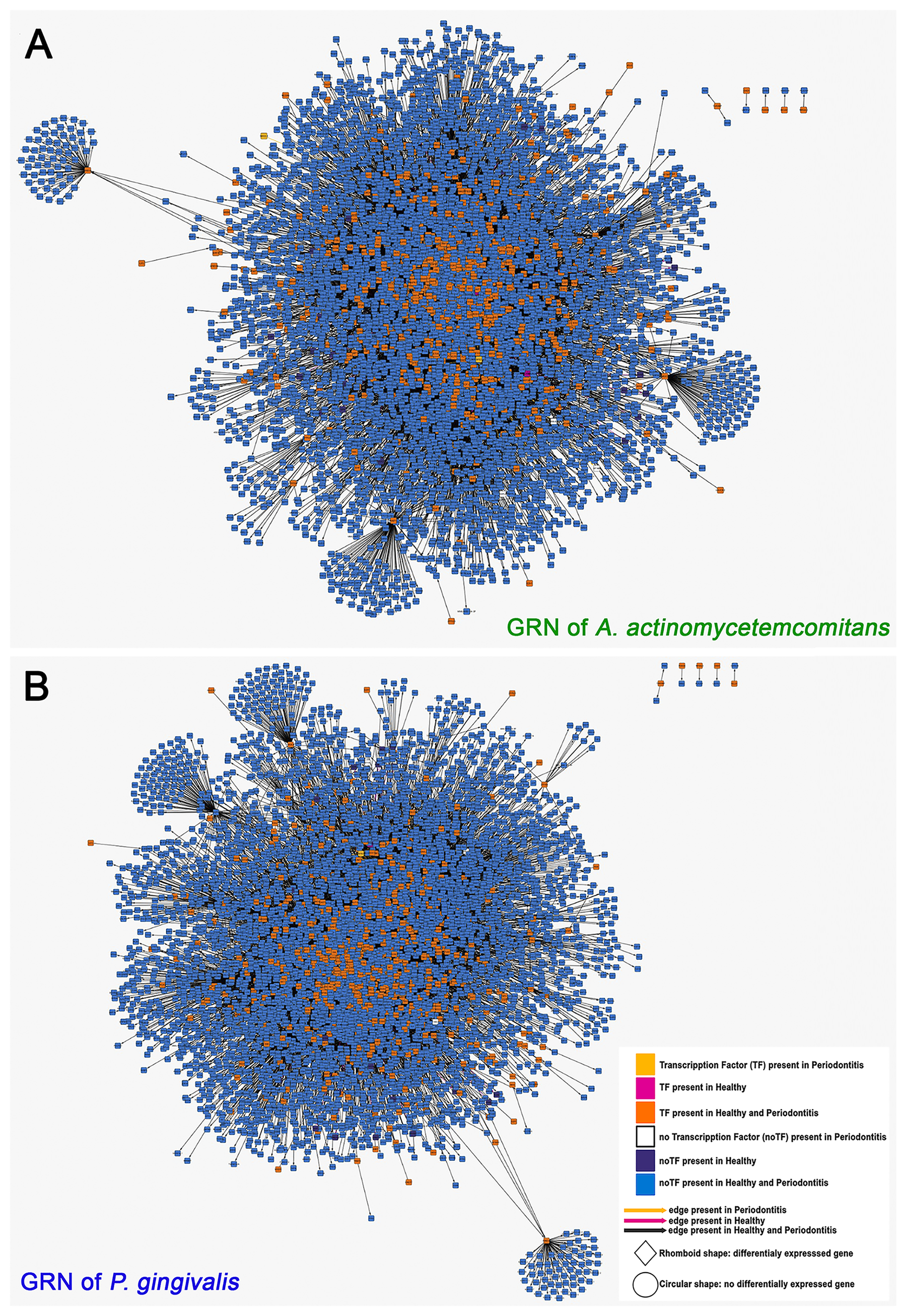

Supplement: Figure S2 — Context-specific networks. [file spectrum.01678-25-s0002.tif]

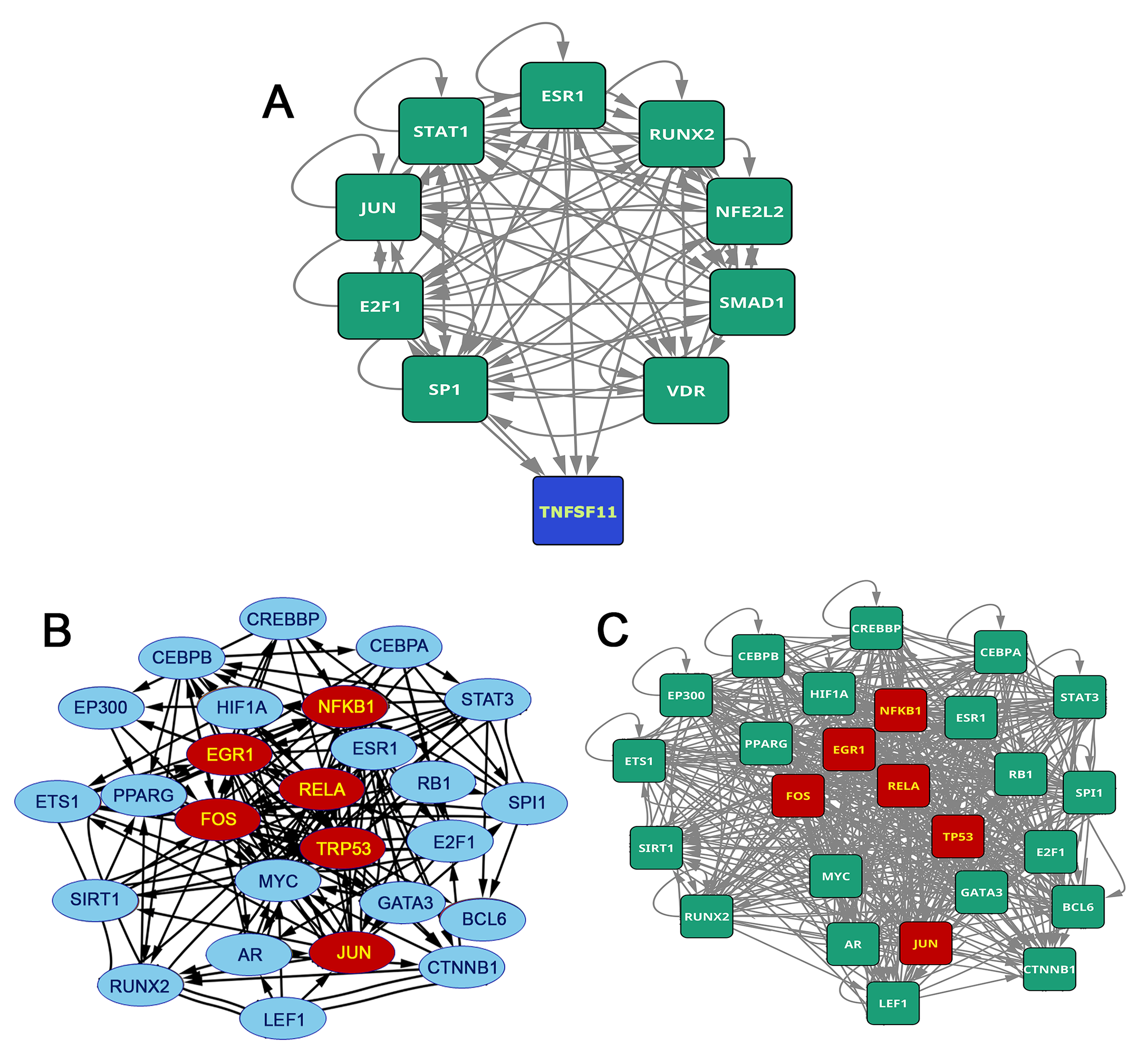

Supplement: Figure S3 — Regulatory reference networks. [file spectrum.01678-25-s0003.tif]
