# Supplementary material for: Transcription-based comparison of Aggregatibacter actinomycetemcomitans or Porphyromonas gingivalis-induced experimental periodontitis
Source: Microbiol Spectr. 2026 Jan 8;14(2):e01678-25. doi: 10.1128/spectrum.01678-25 (PMC12889031; doi:10.1128/spectrum.01678-25)
Supplement: Supplemental legends — Descriptive legends for Fig. S1 to S3. [file spectrum.01678-25-s0004.docx]

**Supplementary figures captions**

**FIG S1** The bar graphs on the left present the ten most overrepresented functional terms. On the right, interaction plots highlight the key genes within each co-expression module. Overrepresentation analysis (-log10 adjusted p-value) was performed using Gene Set Enrichment Analysis from Msigdb [PMID: 16199517] for modules M3 **(A)**, M5 **(B)**, M6 **(C)**, and M7 **(D)** via the CEMiTool and String databases.

**FIG S2** Gene Regulatory Network of *Aa* **(A)**. The network compares context-specific networks under conditions of *Aa* inoculation-induced periodontitis and in the absence of periodontitis (uninfected control). It comprises 6,158 nodes, of which 720 are TFs and 18,683 edges. **B)** Gene Regulatory Network of *Pg*. The network compares context-specific networks under conditions of periodontitis induced by *Pg* inoculation and in the absence of periodontitis. It comprises 6,159 nodes, of which 718 are TFs and 18,612 edges. In both GRNs, the shapes and colors of nodes and edges indicate whether they are associated with periodontitis or healthy conditions.

**FIG S3** Regulatory subnetwork controlling RANKL (TNFSF11) expression mapped onto the human reference TFlink network, highlighting the transcription factors and regulatory interactions that comprise the central regulatory axis driving activation of this key gene in the osteoinflammatory response (S3A). Subnetwork of master regulator genes identified in ligature-induced experimental periodontitis (19). The network comprises 26 master regulators, with a central core (red nodes) formed by the six MR-TFs identified in this study (S3B). This nodal and hierarchical organization is preserved when mapping the subnetwork onto the reference human regulatory network, TFlink (S3C), thereby reinforcing the translational relevance of these central regulators.
